# ZnO/CeO_2_ Nanocomposites: Metal-Organic Framework-Mediated Synthesis, Characterization, and Estimation of Cellular Toxicity toward Liver Cancer Cells

**DOI:** 10.3390/jfb13030139

**Published:** 2022-09-02

**Authors:** Toqa Alabyadh, Riyadh Albadri, Ali Es-haghi, Mohammad Ehsan Taghavizadeh Yazdi, Narges Ajalli, Abbas Rahdar, Vijay Kumar Thakur

**Affiliations:** 1 Department of Biology, Mashhad Branch, Islamic Azad University, Mashhad 91871-47578, Iran; 2 Applied Biomedical Research Center, Mashhad University of Medical Sciences, Mashhad 91388-13944, Iran; 3 Department of Chemical Engineering, Faculty of Engineering, University of Tehran, Tehran 14179-35840, Iran; 4 Department of Physics, University of Zabol, Zabol 98613-35856, Iran; 5 Biorefining and Advanced Materials Research Center, Scotland’s Rural College (SRUC), Edinburgh EH9 3JG, UK; 6 School of Engineering, University of Petroleum and Energy Studies (UPES), Dehradun 248007, Uttarakhand, India; 7 Centre for Research and Development, Chandigarh University, Mohali 140413, Punjab, India; 8 Department of Biotechnology, Graphic Era Deemed to be University, Dehradun 248002, Uttarakhand, India

**Keywords:** zinc oxide, cerium oxide, metal-organic framework, nanoceria, anticancer, doping

## Abstract

The Zinc-doped cerium oxide nanocomposite (ZnO/CeO_2_ NC) was synthesized using a metal-organic framework as a precursor through the combustion method. It was characterized by powder X-ray diffraction (PXRD), Fourier transform infrared spectroscopy (FTIR), field emission electron microscopy (FESEM), energy dispersive analysis (EDX), transmission electron microscopy (TEM), dynamic light scattering (DLS), and ξ-potential. The PXRD demonstrated the successful synthesis of ZnO/CeO_2_ NC with a crystallite size of 31.9 nm. FESEM and TEM images displayed hexagonal and spherical morphologies, and the solid-phase size was 65.03 ± 30.86 nm for ZnO/CeO_2_ NCs. DLS, TEM, and FESEM showed that the NCs have a high tendency for agglomeration/aggregation in both aqueous media and solid phase. The anticancer attributes of ZnO/CeO_2_ NC were investigated against Liver cancer cells (HepG2), which showed inhibition of cancer cell growth on a concentration-dependent gradient. The cell toxicity effects of ZnO/CeO_2_ nanocomposites were also studied toward NIH-3T3, in which the data displayed the lower toxicity of NC compared to the HepG2 cell line.

## 1. Introduction

Nanocomposites (NCs) are an advanced kind of recognized composite material that have been reinforced with nano-sized particles. Such materials could be improved to be appropriate for biomedical uses, of which notable developed material properties are needed, meaning that the material properties of NCs are greater because of the superior features of materials in the nanoscale. NCs are being increasingly used by researchers in medical and pharmacological applications. Hence, it is of excessive importance to be aware of the studies conducted in this area by scientists to be able to predict the behaviors of structures consisting of NCs in in vitro and in vivo conditions.

Nanotechnology can lead to new attributes of matter that can be used in new cutting-edge systems or industries with more efficiency, especially in biological, medical, and pharmacological applications [1,2,3]. Among inorganic nanoparticles, cerium oxide nanoparticles (CeO_2_ NPs) demonstrated a higher catalytic performance due to their higher surface area or exposed surface. The interesting redox characteristics of Ce^3+^/Ce^4+^ are the main cause of its catalytic properties. The bandgap energy reported for CeO_2_ NPs is 3.19 eV [4], which has a moderate exciton binding energy and could well absorb UV radiation. As the catalytic performance of CeO_2_ NPs increase, it can be expected that the anticancer efficacy of CeO_2_ NPs can be increased using other metals or metal oxides as dopants [5]. To achieve the highest catalytic performance, the bandgap energy should be optimized to increase therapeutic efficiency. In some cases, ZnO doping increased the catalytic activity of CeO_2_ in CO oxidation [6], gas sensor performance [7], catalytic operating life [8], and photocatalytic performance [9]. It appears that the better performance of the nanocomposites may be due to changes in the concentration of reactive oxygen species (ROS), for instance hydroxyl radical and superoxide anions [10,11]. It appears that ZnO/CeO_2_ NCs are interesting materials that should be investigated for biological applications, such as anticancer and antibacterial ones. Presently, just a few reports investigate the anticancer or biological effects of ZnO-doped CeO_2_ NPs [12]. Zinc is a common dopant that is used to modify and enhance the properties of other oxides, such as CeO_2_, which could lead to structural changes and better optical, catalytic, or electrical attributes [13,14]. Other factors that Zn dopant could increase are the oxygen storage and thermal stability of the cerium oxide nanocomposites [15]. Therefore, the use of zinc as a dopant could increase the efficiency and add some new characteristics, structures, morphologies, and applications.

The toxicity of nanostructures should be evaluated as a requirement for their safe and effective use in any industry. An in vitro cell toxicity test is one of the tests that can express the toxicity and safety of produced nanoparticles [16]. The aim of nano-toxicity, which also mentions the nano-safety of biomaterials, is to establish a dose–effect association [17]. Also, nano-toxicology contains the study of the interactions of nanomaterials with cells and the study of the potential causes of toxicity from nanomaterials. Indeed, the toxicity of NPs has already been widely considered in environmental pollution [18].

Different synthetic methods, such as hydrothermal, co-precipitation, sol-gel, and combustion, are available [19,20]. In the hydrothermal method, the autoclave vessel is expensive, monitoring is impossible, and safety issues usually exist at higher temperatures [21]. The sol-gel method also may not be affordable, and the large shrinkage of the volume and cracking during the drying process is an issue that may affect the final product [22]. Using metal-organic precursors elevates the purity of final nanoparticles, which is similar to the co-precipitation method. The particle sizes that use metal-organic frameworks as precursors were evenly distributed and can be controlled using different linkers and MOFs structures. The combustion method, which uses a facile synthesized precursor, could be of great use; for instance, metal-organic precursors may include coordination polymers or MOFs [23,24]. Metal oxide nodes in MOFs could form cores to prepare nanoscale metal-oxides by pyrolysis [25,26]. UiO-66 is one of the known MOFs that is applied in different applications of science, but it can also be used as a precursor to synthesize metal oxides [27,28]. The linker, 1,4-benzene dicarboxylic acid, is an available and affordable organic precursor. Different metal precursors, such as Ce, Cu, Co, Mn, Fe, Zr, and Hf, can be used to prepare the crystalline framework [29,30,31]. One of the most important factors that affects the purity of the final nanoparticle is the crystallinity of the MOFs [31]. The other one could be the formation of uniform particle sizes with a specified morphology and hierarchical superstructures with high surface areas [25]. In addition, catalytic studies also indicate that CeO_2_ NCs and NPs that are prepared using UiO-66 _Ce_ showed more catalytic activity, stability, and selectivity compared to commercial CeO_2_ [32,33]. Therefore, the synthesis of CeO_2_ NCs by applying MOFs may enhance anticancer activities because biological effects can directly relate to the catalytic properties of the prepared metal oxide and nanocomposites.

In this study, the anticancer properties of the newly-synthesized ZnO/CeO_2_ NCs were investigated, in which a coordination polymer (UiO-66 _Ce-Zn_) was used as a precursor. Herein, zinc was doped into the MOF before synthesis, along with other precursors. As it was mentioned, MOFs were applied in the synthesis of metal oxide nanoparticles, but the application of dopants (trace of other metals) without changing the structure could widen their use in the synthesis of different and more active nanoscale mixed metal oxides. Therefore, the synthesis of the MOF was the same as the previous report in the literature [34], and only zinc salt was used as a dopant to be used as a precursor for the synthesis of ZnO/CeO_2_ NCs. Due to the importance of the physicochemical characteristics of ZnO/CeO_2_ NCs, the phase purity, crystallite size, functional groups, morphology, solid-phase size, hydrodynamic size, and surface charge were fully determined and discussed using conventional methods such as PXRD, FTIR, FESEM, TEM, DLS, and ξ-potential. Most importantly, our study investigated the biological functions of the prepared NCs. To the best of our knowledge, the cytotoxicity of ZnO/CeO_2_ NCs was performed for the first time on NCs obtained from the pyrolysis of UiO-66 _Ce_ and zinc as a dopant. A liver cancer cell line (HepG2) was used to measure the anticancer potential of ZnO/CeO_2_ NCs.

## 2. Materials and Methods

### 2.1. Materials

All the materials were procured from Merck and Sigma chemical group unless otherwise stated. The cancer cell lines were obtained from the Pasteur Institute of Iran.

### 2.2. The Synthesis of the ZnO@CeO_2_ NCs

**Figure 1:**
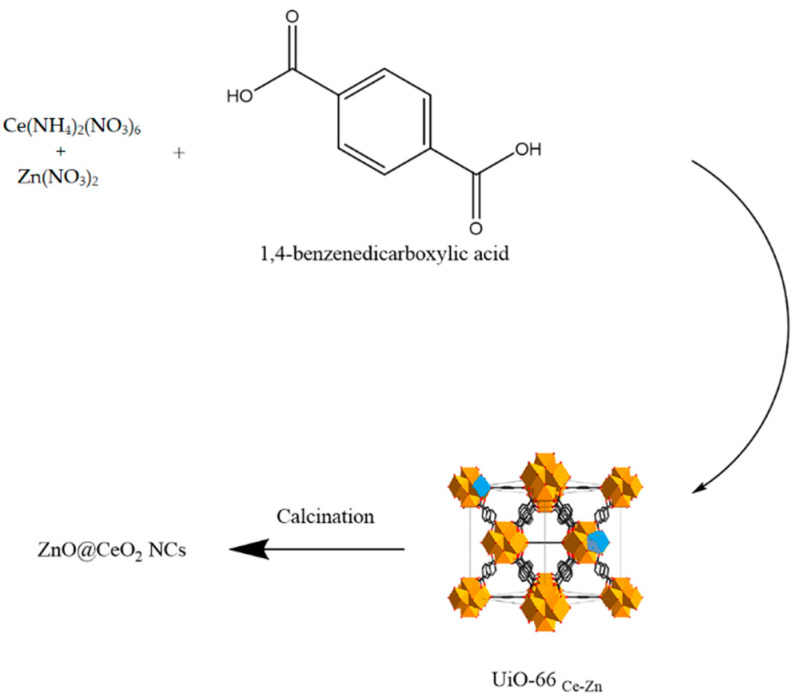
Pictorial diagram for synthetic procedure.

The precursor (UiO-66 _Ce-Zn_) was prepared as it was explained in the literature for the synthesis of UiO-66 _Ce_ and used with no further purification (35). In detail, 2000 mg of benzene dicarboxylic acid was dissolved in 368 mL of DMF. Then, 6604 mg of Ce(NH_4_)_2_(NO_3_)_6_ and 360 mg of Zn(NO_3_)2·6H_2_O were dissolved in DMF and mixed with the first solution. Then, it was stirred at 100 °C for 20 min and the precipitate was separated. It was washed with DMF three times and dried at 80 °C overnight. After that, the precursor (4 g) was calcined at 600 °C for 5 h (Figure 1).

### 2.3. Characterization

ZnO/CeO_2_ NCs were studied by PXRD (Advance-Bruker, Germany), in which the sample in powder form was analyzed from 2theta values in the range of 10–70° using Cu-radiation. The nanocomposites were scrutinized using FTIR spectroscopy by a Shimadzu8400 device, where KBr pellets of the samples were made and FTIR was set from the 400 to 4000 cm^−1^ region. TEM was performed using a ZEISS LEO 912 AB. The sample was dispersed in water using ultrasound waves, then one drop of the suspension was poured onto a copper plate and dried and used for TEM analysis. For FESEM, a TESCAN device (MIRA 3) was used, and the sample was in powder form. DLS was performed using a Particle Size Analyzer, along with Vasco3 and Zeta potential. For DLS and Zeta potential analyses, 2 mg of the sample were dispersed using ultrasound waves in 10 mL of distilled water at a pH of 7.

### 2.4. In Vitro Cell Toxicity

A human liver carcinoma cell line (HepG2) was selected as an appropriate in vitro model. HepG2 cells were attained from Pasteur Institute, Iran. The cytotoxicity effects of NPs against HepG2 cells were evaluated by the MTT assay. The cells were seeded in 96-well plates (5000 cells per well) and incubated overnight at 37 °C Then, the cells were treated with different concentrations of NPs for 24, 48, and 72 h. After these times, the treatment medium was drained and 100 μL of MTT solution (0.45 mg/mL) was added to each well and incubated for 4 h to form a formazan crystal. The insoluble formazan crystals were then dissolved in DMSO and shaken for 20 min. Finally, the absorption of the samples was recorded using a plate reader (Stat fax 2100, USA) at 570 nm with the reference wavelength of 630 nm. The cell viability was calculated using the following formula:

Cell viability (%) = (OD treated cells /OD un-treated cells) × 100

### 2.5. Statistical Analysis

With the purpose of estimating the toxicological activity of the nanocomposite and comparing it with that of the normal cell line sample, the resultant values were moved to SPSS software. One-way ANOVA was employed to compare the means by LSD. Error-bar values and a 5% confidence level were used for calculations.

## 3. Result and Discussion

### 3.1. Fourier Transform Infrared Spectroscopy (FTIR)

**Figure 2:**
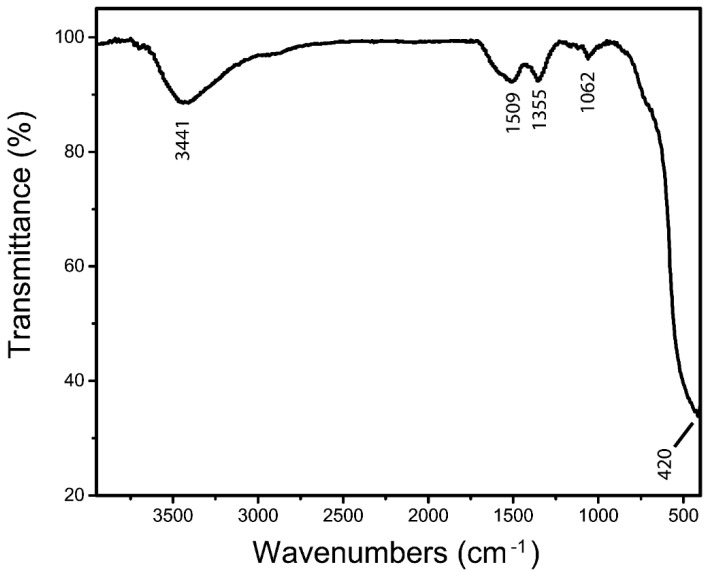
The FTIR spectrum of the ZnO/CeO_2_ NCs.

The FTIR spectrum of the ZnO/CeO_2_ NCs from 400 to 4000 cm^−1^ demonstrated the presence of the functional groups (Figure 2). The band at 3441 cm^−1^ and 1509 cm^−1^ corresponded to stretching and bending vibrations of O-H groups or the absorbed H_2_O [35,36]. The bands, including 1355 cm^−1^, and 1062 cm^−1^, could be related to residual organic compounds [37], and the band observed at 420 cm^−1^ could be recognized as a Ce-O vibration [38,39].

### 3.2. Powder X-ray Diffraction (PXRD)

**Figure 3:**
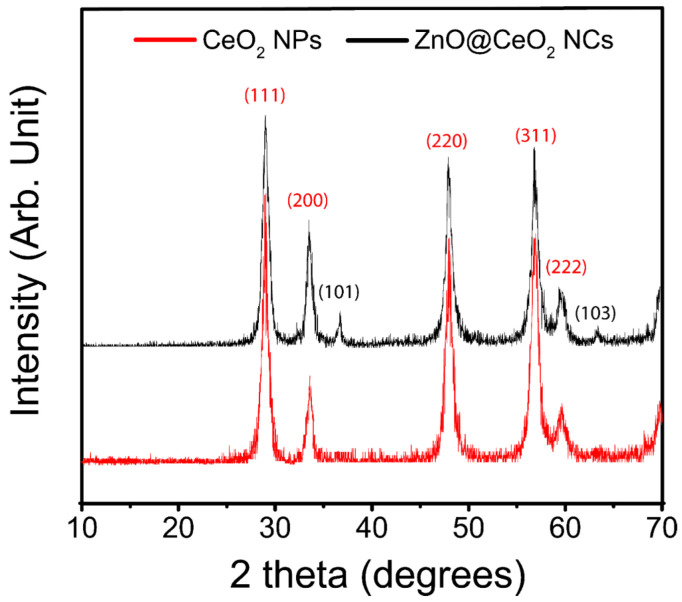
PXRD analyses of the CeO_2_ NPs and ZnO/CeO_2_ NCs.

The PXRD has shown structural changes after the addition of zinc dopant (Figure 3). The multi-phase compound of CeO_2_ and ZnO were consistent with the JCPDS numbers of 01-081-0792 and 01-079-0208, respectively. The crystal system, space group, and space group number of nanoceria were cubic, Fm-3 m, and 225, respectively. The calculated 2theta values and intensities were 28.5° (100%), 33.1° (28.5%), 47.5° (45.8%), 56.3° (36.1%), and 59.1° (7.1%), which corresponded to the HKL of (1 1 1), (2 0 0), (2 2 0), (3 1 1), and (2 2 2), respectively. The experimental values and calculated ones were consistent with each other, which showed the purity of the samples. The 2theta values and HKLs of 36.45° (1 0 1) and 63.44° (1 0 3) in ZnO/CeO_2_ NCs were associated with the ZnO, and it appears no other impurities were found in the sample. The crystallite sizes were obtained by the Scherrer equation, which was 26.6 nm and 31.9 nm for nanoceria and ZnO/CeO_2_ NCs, respectively.

### 3.3. Field Emission Scanning Electron Microscopy (FESEM)

**Figure 4:**
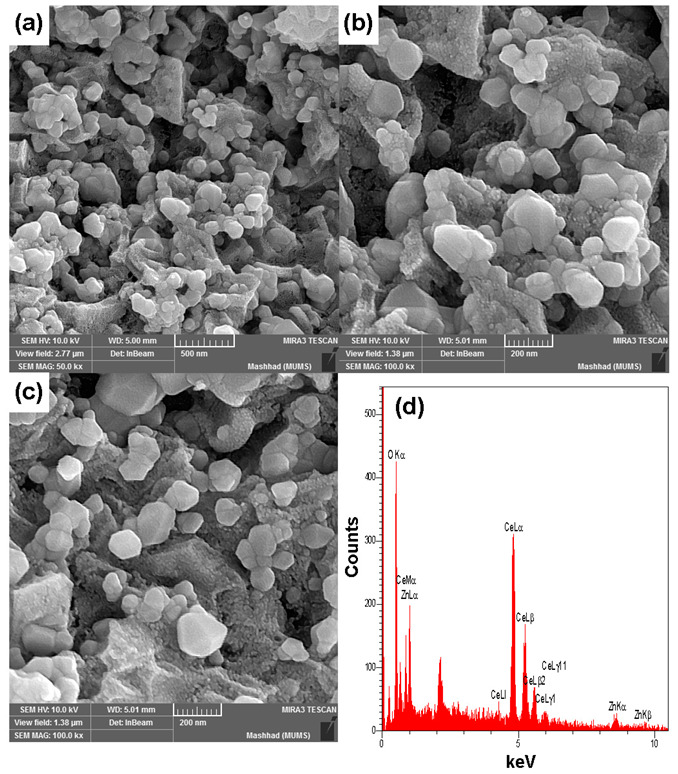
(**a**–**c**) FESEM images and (**d**) EDX analysis of the ZnO/CeO_2_ NCs.

The FESEM images demonstrated hexagonal, spherical, and shapeless morphologies for ZnO/CeO_2_ NCs (Figure 4). The images indicated high aggregation/agglomeration compared to the crystallite sizes. The EDX analysis also confirmed the elemental composition of ZnO/CeO_2_ NCs. The Ce Lα and Zn Kα were associated with cerium and zinc, respectively, which were at 4.8 keV and 1.0 keV accordingly.

### 3.4. Transmission Electron Microscopy (TEM)

**Figure 5:**
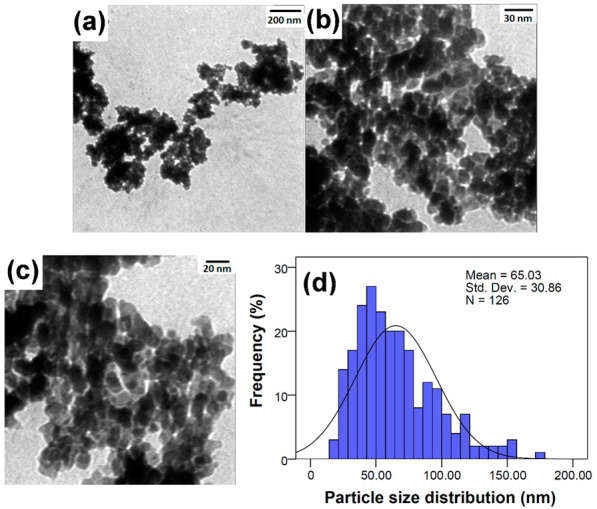
(**a**–**c**) FESEM images and (**d**) particle size distribution of the ZnO/CeO_2_ NCs.

The TEM images (Figure 5) showed that the ZnO/CeO_2_ NCs were spherical or semi-spherical particles with a diameter of 65.03 ± 30.86 nm. Compared to the crystallite size (31.9 nm), the TEM images demonstrated high agglomeration in the sample. The results were similar to the results from the FESEM images.

### 3.5. Dynamic Light Scattering (DLS) and ξ-Potential

**Figure 6:**
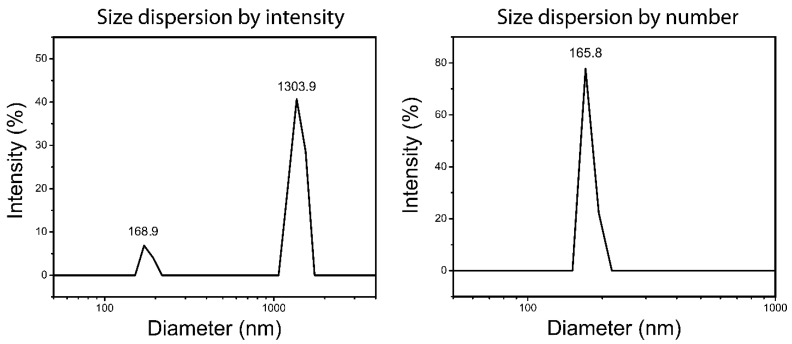
The hydrodynamic size dispersion of the ZnO/CeO_2_ NCs by intensity and number.

The DLS analysis demonstrated (Figure 6) highly-agglomerated particles in ZnO/CeO_2_ NCs. Compared to the solid-phase diameter (65 nm) and crystallite size (31.9 nm), the hydrodynamic size dispersion by intensity showed a bimodal pattern with two diameters (168.9 ± 10.1 nm and 1303.9 ± 115.3 nm) that indicated a broad distribution of particles. The Z-average and polydispersity were 919.7 nm and 0.6, respectively. Although size dispersion by number showed a diameter of 165.8 ± 8.7 nm, the intensity-based size dispersion indicated that the flocculation was similar the TEM results. The tendency for the flocculation of particles may be due to hydrogen bonds or van der Waals forces [40,41]. The ξ-potential was −24.8 mV, which could confirm the incipient instability and floc formation [42].

### 3.6. Cell Toxicity Effect of ZnO/CeO_2_ NCs towards HepG2

**Figure 7:**
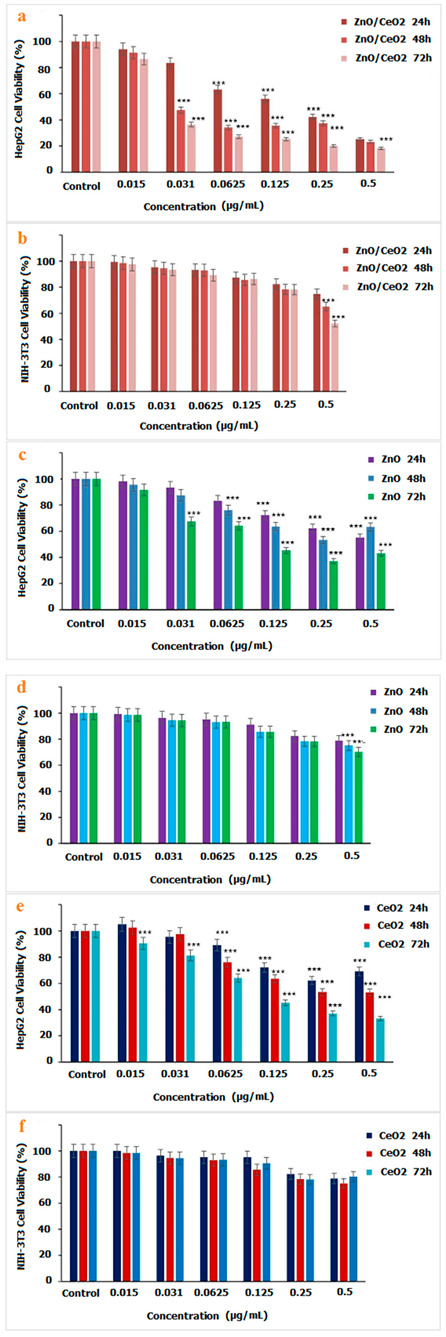
Comparative diagram of cytotoxicity properties of fabricated (**b**) ZnO/CeO_2_, (**d**) ZnO, (**f**) CeO_2_ NCs against NIH-3T3 and cytotoxicity properties of (**a**) fabricated ZnO/CeO_2_, (**c**) ZnO, (**e**) CeO_2_ NCs against HepG2. The percentages were displayed relative to control cells. * *p* < 0.05, ** *p* < 0.01, *** *p* < 0.001 indicated significant difference as compared to the control.

**Figure 8:**
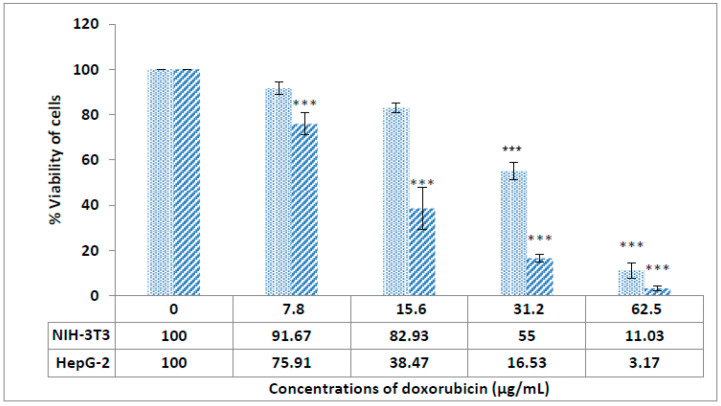
The cytotoxicity effect of doxorubicin against NIH-3T3 and HepG2. ** *p* < 0.01, *** *p* < 0.001 indicated significant difference as compared to the control.

To assess the anticancer activity of the ZnO/CeO_2_ nanocomposites, diverse concentrations of NC were added to liver cancer cell lines (HepG2), and the cytotoxicity consequences were tested at 24, 48 and 72 h. The consequences revealed that there is a gradual increase in cell death when enhancing the concentration of ZnO/CeO_2_ NCs (Figure 7). A comparative study of cytotoxicity in ZnO, CeO_2_, and ZnO/CeO_2_ NCs showed that the formation of ZnO/CeO_2_ NCs could lead to improved cytotoxicity results against cancer cells. As stated by the WHO, cancer is the second leading cause of death in the world [43]. Conventional cancer cures depend on surgical procedures and chemotherapy. Oftentimes, mixtures of these treatments are necessary to wholly wipe out the disease. However, normal tissues are affected by these actions as well, thereby leading to contrary side effects [44]. Nanomedicine can support conventional cures for cancer via several biocompatible nano-raised areas [45,46,47]. ZnO is inexpensive and is used in cosmetics and skin-care goods, such as sunscreens, because it absorbs UV light [48]. Several reports indicate that ZnO nanoparticles have a key role in biomedical applications [49,50]. Particularly, ZnO NPs have also been identified because they influence a lot of cancer cells in vitro [51], given that ZnO NPs activate ROS fabrication and consequently lead to cancer cell death [52,53]. Cerium oxide nanoparticles have an extensive variety of uses in diverse areas such as biology and therapeutic sciences [54]. There are various reports that nanocrystals have an inhibitory effect against various cancer cell lines and, at the same time, have low cytotoxicity in normal cells [55]. The DNA destruction of cancer cells by the formation of hydroxyl groups and inhibition of NF-kB protein, which has a significant role in cancer progression, are important mechanisms of nanoceria against cancerous cell lines [56]. According to investigations, during liver regeneration, it is important to preserve the proliferative state until the original liver mass is restored [57]. However, in several liver disorders, the accumulation of ROS may inhibit ideal regeneration because of the induction of apoptosis due to lipid peroxidation, consequently inhibiting the resolution of tissue destruction. ROS act as mediators in the regulation of diverse growth agents, transcription factors, and cell cycle proteins‚β-catenin, cyclin D, p53, and NF-E2-related factor 2 (Nrf2), for example. All of these proteins are necessary for the regenerative process, and improper regulation results in detrimental properties in liver regeneration. It has been shown that the presence of ZnO NPs in cancer cells causes an increase in the production of caspase 3 due to the increase of ROS, which ultimately causes apoptosis and cancer cell death [58]. Cell viability studies showed that doxorubicin-loaded polymerized magnetic nanocarriers could lead to high death rates among HepG2 liver cancer cells [59]. Cerium in nanoform causes increased toxicity in human HuH-7 cells due to damage to their DNA [60]. CeO_2_NPs modified the messenger expression of pro-inflammatory and oxidative stress-related genes, including iNOS, myeloperoxidase (MPO), prostaglandinendoperoxide synthase 1 (PTGS1), and neutrophil cytosol factor 2 (Ncf2), and these coppers can play a role in improving liver function [57]. The simultaneous use of ZnO and CeO NPs can help to improve the treatment of liver cancer and result in better functioning in the liver. A decrease in cell viability was detected when the DOX was introduced into the cells (Figure 8). These consequences also showed that enhancing the drug concentration and exposure time resulted in a reduction in the cell viability of cancerous cells.

## 4. Conclusions

The ZnO/CeO_2_ NCs were prepared by the combustion method using a metal-organic framework as a facile synthesized precursor, especially in metal-doped metal oxides. The NCs were fully characterized, and their physicochemical factors showed the successful synthesis of ZnO/CeO_2_ NCs. It appears that the NCs demonstrated an inclination toward flocculation. The anticancer attributes of samples also indicated the lethal effect of NCs on liver cancer cell lines, which is appropriate for further use in biomedical fields.
